# Two Classifiers Based on Serum Peptide Pattern for Prediction of HBV-Induced Liver Cirrhosis Using MALDI-TOF MS

**DOI:** 10.1155/2013/814876

**Published:** 2013-02-19

**Authors:** Yuan Cao, Kun He, Ming Cheng, Hai-Yan Si, He-Lin Zhang, Wei Song, Ai-Ling Li, Cheng-Jin Hu, Na Wang

**Affiliations:** ^1^Department of Laboratory Medicine, Jinan Military General Hospital, Jinan, Shandong 250031, China; ^2^Institute of Basic Medical Sciences, National Center of Biomedical Analysis, Beijing 100850, China; ^3^Department of Neurology, Jinan Military General Hospital, Jinan, Shandong 250031, China

## Abstract

Chronic infection with hepatitis B virus (HBV) is associated with the majority of cases of liver cirrhosis (LC) in China. Although liver biopsy is the reference method for evaluation of cirrhosis, it is an invasive procedure with inherent risk. The aim of this study is to discover novel noninvasive specific serum biomarkers for the diagnosis of HBV-induced LC. We performed bead fractionation/MALDI-TOF MS analysis on sera from patients with LC. Thirteen feature peaks which had optimal discriminatory performance were obtained by using support-vector-machine-(SVM-) based strategy. Based on the previous results, five supervised machine learning methods were employed to construct classifiers that discriminated proteomic spectra of patients with HBV-induced LC from those of controls. Here, we describe two novel methods for prediction of HBV-induced LC, termed LC-NB and LC-MLP, respectively. We obtained a sensitivity of 90.9%, a specificity of 94.9%, and overall accuracy of 93.8% on an independent test set. Comparisons with the existing methods showed that LC-NB and LC-MLP held better accuracy. Our study suggests that potential serum biomarkers can be determined for discriminating LC and non-LC cohorts by using matrix-assisted laser desorption/ionization time-of-flight mass spectrometry. These two classifiers could be used for clinical practice in HBV-induced LC assessment.

## 1. Introduction

Patients with liver cirrhosis (LC) caused by chronic hepatitis B virus (HBV) are at high risks of developing hepatocellular carcinoma (HCC) [1–4]. In China, the proportion of people infected with HBV is higher than that in other countries with an estimated 120 million [5, 6]. During a 5-year period, 10%–20% of patients with chronic hepatitis developed cirrhosis, and 6%–15% of the people with cirrhosis and chronic hepatitis progressed to HCC, among whom 5-year survival is less than 5% [7]. At present, liver biopsy has been the “gold standard” for evaluation of stage of liver fibrosis and cirrhosis [8], but it is limited as it is an invasive procedure with significant expense, manpower issues, and some risks. Furthermore, intra- and interobserver variations for interpretation of biopsies are 10%–20%, even among experienced pathologists [9]. For this reason, developing diagnosis biomarkers of LC is an alternative way for assessing prognosis and candidacy for treatment in patients with chronic liver disease. Over the past decade, attempts have been made to develop noninvasive methods to assess LC, including physical approaches and biological approaches. Physical approaches include 2-D acoustic radiation force impulse imaging (ARFI), 3-D magnetic resonance (MR) elastography, and 1-dimensional ultrasound transient elastography (TE) [10]. ARFI can be easily implemented, but it has a limited range compared with TE [11]. TE analysis has excellent inter- and intraassay agreements, but its applicability (80%) is not as good as that of serum biomarkers [12]. Although MR elastography can analyze almost the entire liver, it is too expensive and time consuming to use in routine practice [8]. In recent years, serum-based tests of liver cirrhosis have attracted more attention, such as the aspartate to platelet ratio index [13] and the FibroTest [8, 14–18]. However, most of these studies on biomarkers of liver cirrhosis have been conducted in chronic hepatitis C, and few data are available on the applicability of this approach to patients infected with HBV [19]. Meanwhile, some serum biomarkers related to the fibrogenic process, such as hyaluronic acid, may be confounded by associated diseases with fibrosis in other organs [20].

Recently, proteomics studies using high-throughput spectrometric methods such as matrix-assisted laser desorption/ionization time-of-flight mass spectrometry (MALDI-TOF MS) and surface-enhanced laser desorption/ionization time-of-flight mass spectrometry (SELDI-TOF MS) have proved possible methods for the identification of new disease biomarkers [21]. Up to now, advances based on proteomics have been made in the understanding of hepatitis and liver cirrhosis. Zhu et al. [22] proposed two serum biomarkers for HBV-induced LC using SELDI technology. They obtained a sensitivity of 80.0% for all LC patients and a specificity of 81.8% for all noncirrhotic cohorts. Bozdayi et al. [15] provided similar results with a sensitivity of 83.3% and a specificity of 85.1%. However, establishment of serum peptide pattern for predicting HBV-induced LC from noncirrhotic cohorts remains challenging. Consequently, the objective of this work was to identify serum peptidome signatures associated with liver cirrhosis by using the MALDI-TOF MS and to construct classifiers for predicting liver cirrhosis in patients with HBV infection.

## 2. Materials and Methods

### 2.1. Patients and Sample Collection

From December 2009 to August 2010, a total of 162 serum samples including 44 LC patients with chronic hepatitis B (CHB), 46 patients with CHB, and 72 healthy individuals were collected with informed consent in Jinan Military General Hospital (Jinan, China). The design of the study was approved by the Hospital Ethical Committee. Two groups of consecutive subjects were enrolled. The first group consisted of patients with HBV-infected LC. LC patients were diagnosed mainly depending on clinical history, physical examination, laboratory results, and ultrasonographic and/or computed tomographic imaging with liver biopsy [23]. The second group consisted of patients with HBV and healthy individuals. Patients with HBV were diagnosed based on HBsAg (+), HBeAg (+), and HBVDNA (+), as well as abnormal liver biochemistry. Healthy individuals showed no risk factors for viral hepatitis, no history of liver disease, and normal liver function.

Blood sampling from patients was done before the initiation of specific therapy. The blood samples were collected in 5 mL BD Vacutainers without anticoagulation and allowed to clot at room temperature for up to 1 hr [24]. The sera were obtained by centrifugation at 2000 rpm for 15 min and were immediately frozen and stored at −80°C until testing. It took no more than 60 min from serum collection to frozen storage.

### 2.2. Peptide Separation and MALDI-TOF MS Analysis

Copper-chelated magnetic beads and solutions were used for extracting peptides from serum samples [25]. In brief, 5 *μ*L serum was mixed with 5 *μ*L beads and 20 *μ*L of binding solution. The beads were washed three times with 100 *μ*L of wash solution after 10 min incubation [26]. Then, 20 *μ*L of eluent buffer were used to elute the bound peptides. After mixing with 1 *μ*L of CHCA matrix solution, the eluent was spotted onto a 600 *μ*m-diameter spot size 384 MTP target plate (Bruker Daltonik, Germany) until dry. The peptide calibration standard was applied to target spots for external calibration of the instrument in the same matrix. To obtain the peptidome of serum samples, the analysis of the processed samples was performed by an Autoflex MALDI-TOF MS (Bruker Daltonics, Germany) equipped with a pulsed ion extraction ion source.

Serum pools were analyzed from healthy volunteers 5 times for reproducibility. Within-run assays were carried out by using MALDI-TOF MS. The mean value and coefficient of variance (CV) were calculated, respectively.

### 2.3. Data Preprocessing and Feature Selection

Three bioinformatics tools were employed for data processing and analysis, including ClinProt Tools software 2.2 (Bruker Daltonik, Germany), flexAnalysis (Bruker Daltonics, Germany), and Waikato Environment for Knowledge Analysis (WEKA) [27]. First, mass spectra of all serum sample data derived from LC and control were preprocessed via the ClinProt, including baseline subtraction of spectra, normalization and recalibration of a set of spectra, and internal peak alignment by using prominent peaks. Then, the processed data were stored in BioSunMS [28] and were prepared for feature selection in WEKA. In BioSunMS, independent training set (*n* = 81) and the test sets (*n* = 81) were created. Based on the training set, peak statistics were done by the SVM-based strategy in WEKA. In feature selection, “cross-validation” was selected as the attribute selection mode, and tenfold splits as default were performed. Finally, we obtained a list of peaks sorted along the statistical difference between two classes (e.g., LC and control), which was used for further data analysis.

### 2.4. Classifier Construction and Evaluation

To predict liver cirrhosis, five classifiers were constructed based on the training set using five supervised machine learning methods (naïve Bayes: NB: multilayered perceptron: MLP: support vector machine: SVM; C4.5 decision tree; DT; classification and regression tree; CART) in WEKA, respectively. A 10-fold cross-validation was performed to avoid model-specific overfitting. To perform cross-validation, all the records were randomly divided into ten parts; nine sets were used for training and the rest one for testing. The process was repeated ten times and the accuracy for true, false and total accuracy was calculated. The final accuracy is the average of the accuracy in all ten tests. With the test set (22 from patients with LC, 23 from patients infected with HBV, and 36 from healthy individuals), we evaluated the generalization performance of the five classifiers by considering the number of correctly classified (true positives, TPs, and true negatives, TNs) and incorrectly classified (false positives, FPs, and false negatives, FNs) samples in the test set. Accuracy (ACC), sensitivity (SE), and specificity (SP) were also calculated. To evaluate the performance of a range of classifiers, StAR [29] was used to plot receiver operator curves (ROCs) and statistical comparison of area under curves. 

## 3. Results

### 3.1. Reproducibility of the Autoflex System

**Figure 1:**
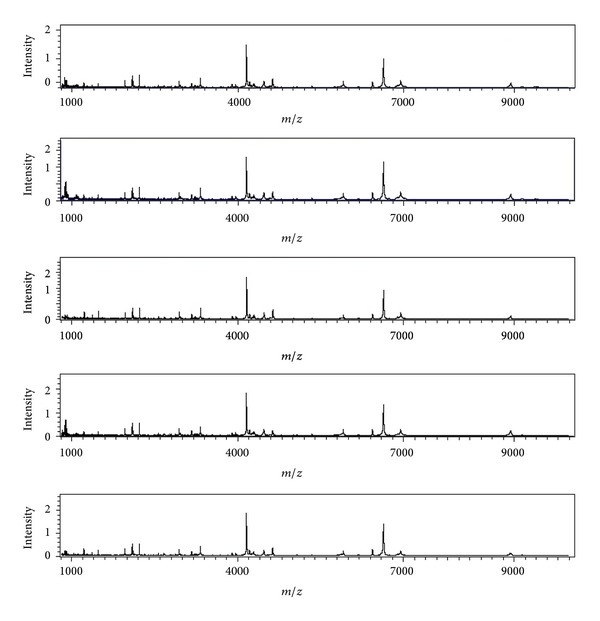
Spectra illustrating reproducibility of 5 separate analyses from controls.

The reproducibility with ten mixed serum samples from the healthy controls was evaluated by the Autoflex MALDI-TOF MS. The mixed serum samples were spotted on five spot WCX magnetic beads. Overall, the Autoflex revealed the mean CV (19.8%) for within-run assay (Figure 1).

### 3.2. Dataset and Feature Selection

**Figure 2:**
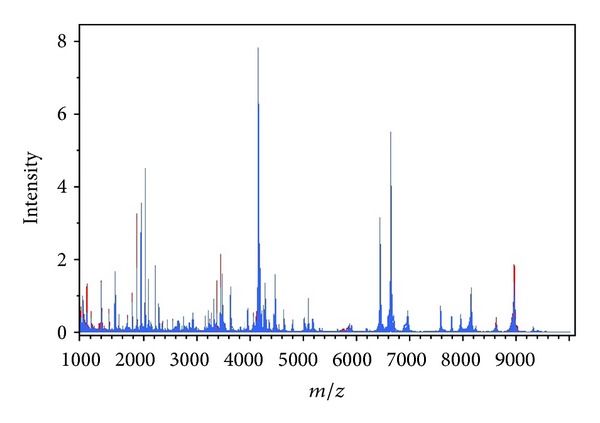
Complete mass spectrum of serum samples between HBV-cirrhosis and non-LC groups in the 800–10,000 *m/z* range. Red line represents HBV-cirrhosis group; Blue line represents non-LC group.

To screen serum peptides for prediction of liver cirrhosis in chronic hepatitis B infection, we used MALDI-TOF MS to analyze serum samples of 44 LC patients and 118 non-LC individuals (46 HBV infected and 72 healthy). The complete mass spectrum comparison of serum samples between LC and non-LC groups is listed in Figure 2. A total of 235 peaks with *m/z* between 800 and 10000 Da were obtained from the 162 serum samples. Fifty percent of subjects serum spectra were used as training set (*n* = 81) and others as test set (*n* = 81) (Table 1). After data preprocessing, feature variables were evaluated by using the SVM-based strategy and ranked by the square of the weight assigned by the SVM. A panel of 14 peptides was selected for classifier construction based on the training set, which have *m/z* ratios of 807, 916, 1011, 1017, 1449, 1536, 1785, 1928, 2551, 3951, 4202, 4207, 4281, and 6945 Da. Because the samples were from HBV-infected patients with and without LC and healthy individuals, we thus compared the peptide patterns between each among the three groups. With the SVM strategy, the top twenty peptides marked differently between two groups were listed in Table 2. We found that the peptide of 3951 Da just revealed significant difference between HBV without LC and healthy individuals. Therefore, we excluded this peptide from the peptide pattern.

### 3.3. Construction and Evaluation of the Five Classifiers

To predict LC, five classifiers including LC-NB, LC-MLP, LC-SVM, LC-DT, and LC-CART were constructed by five supervised machine learning methods, that is, NB, MLP, SVM, DT, and CART, respectively. To evaluate the performance of the five classifiers, we applied a preliminary test by tenfold cross-validation on the training set (Table 3). LC-MLP brought the best results with ACC of 97.5%, SE of 95.5%, and SP of 98.3%. The ACC, SE, and SP of LC-NB were 93.8%, 86.4%, and 96.6% respectively. LC-SVM indicated the comparable results with ACC of 90.1% and SP of 96.6% but gave the poorer SE of 72.7%. LC-DT and LC-CART displayed similar results on accuracy and specificity (ACC, 86.4%, SE, 68.2%, and SP, 93.2%, for LC-DT, and ACC, 85.2%, SE, 59.1%, and SP, 94.9%, for LC-CART).

**Figure 3:**
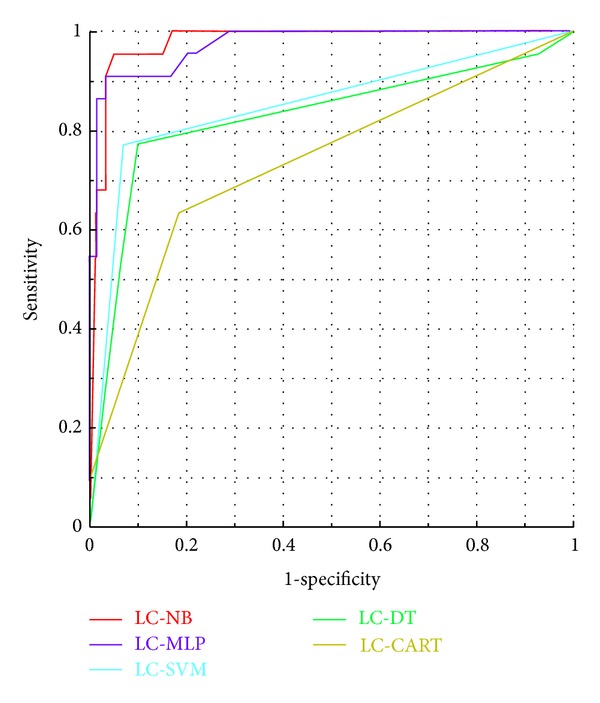
Classifier performances in ROC space. Red line shows ROC curve of LC-NB. Blue line represents ROC curve of LC-MLP. Cyan line represents LC-SVM. Green line represents LC-DT. Yellow line represents LC-CART. The areas under curve are 0.977, 0.973, 0.853, 0.825, and 0.733 for LC-NB, LC-MLP, LC-SVM, LC-DT, and LC-CART, respectively.

The ability of a classifier to discriminate data correctly in the test set is known as its generalization performance [30]. We thus compared the generalization performance of a series of classifiers by plotting their performance on the test set in ROC space (Figure 3). The LC-MLP classifier (Table 4 and Figure 3) showed the best results on the test set, with SE of 90.9% and SP of 94.9% (overall accuracy 93.8%), while LC-NB presented the highest SP of 96.6% (overall accuracy 93.8%). The AUCs are 0.977, 0.973, 0.853, 0.825, and 0.733 for LC-NB, LC-MLP, LC-SVM, LC-DT, and LC-CART, respectively. The difference of AUCs between LC-NB and LC-MLP has no statistical significance at a default significant level (*P* = 0.7871). 

## 4. Discussion

In this study, we obtained thirteen serum peptides for the prediction of HBV-induced liver cirrhosis by statistical comparison of serum peptide pattern of LC and control with MALDI-TOF MS. Subsequently, we investigated five supervised machine learning methods for predicting liver cirrhosis. We found that LC-NB and LC-MLP gave better results (AUC > 0.950) among the five classifiers. Our results indicate that the two classifiers are useful tools for predicting HBV-infected liver cirrhosis through analysis of the serum peptide pattern.

LC is defined as the histological development of regenerative nodules surrounded by fibrous bands in response to chronic liver injury, which is mainly caused by HBV in China [10, 31]. The latest European Association for the Study of the Liver (EASL) treatment guidelines on HBV in 2009 recommend that abnormal ALT levels together with HBV DNA levels of more than 2,000 IU/mL should be evaluated further by liver biopsy [32]. Although histological assessment provides valuable information on the degree of necroinflammation and fibrosis in such patients, it is an invasive procedure associated with a finite albeit small risk of severe complications of 0.5%, patient discomfort, and expense [9]. Therefore, there is a need for a simple, reliable, and noninvasive alternative method for regular monitoring of disease progression. MALDI-TOF MS analysis is a new potential tool for human diseases diagnosis. The low molecular weight proteome or peptidome found in biological fluids can be used to discriminate between patients with or without disease [33]. In addition, combinations of candidate proteins/peptides can often create a more robust test when individual proteins/peptides fail to discriminate between two groups [34]. In this study, we developed a novel approach to predict LC patients infected with HBV based on bead extraction and MALDI-TOF MS analysis. This is a simple, available, and noninvasive procedure compared with other diagnostic approaches. Additionally, the two classifiers, LC-NB and LC-MLP, could differentiate patients with liver cirrhosis from patients with HBV infection or healthy individuals with high sensitivity (86.4%–90.9%) and specificity (94.9%–96.6%). Such accuracy compares favorably with models derived from SELDI or MALDI analysis in similar study. In Zhu's report [22], the researchers were able to predict HBV-induced liver cirrhosis with 80% sensitivity and 82% specificity using SELDI protein chips. Poon and his colleagues [9] built artificial neural network (ANN) fibrosis index for predicting the degree of fibrosis using SELDI ProteinChip arrays. Their accuracy for prediction of cirrhosis reached 89%. Cui et al. [23] obtained a sensitivity of 91.7% and specificity of 94.3% with a SELDI-based serum decision tree classification model from 18 patients with HBV-induced liver cirrhosis and 52 healthy controls. Although their results were better, the HBV-infected patients were not included in the control group. 

Feature selection is an important step of data preprocessing [30]. In this work, we filtered fourteen significantly different peptides between HBV with LC and control (HBV without LC and healthy individuals). Then, we compared the peptide patterns between each among the three groups using the SVM strategy (Table 2). Six peptides from the peptide pattern including 6945, 4202, 807, 1017, 4281, and 1785 Da showed obvious difference between HBV with LC and HBV without LC. Nine of the fourteen peptides selected including 6945, 1928, 916, 807, 1536, 4207, 4281, 1011, 1449, and 2551 Da showed significant difference between HBV with LC and healthy individuals. Among the fourteen peptides selected, the peptides of 6945, 4281, and 807 Da in HBV with LC show higher level than that of HBV without LC and healthy individuals. They may be potential serum biomarkers during LC development in CHB patients. During our analysis, the gender was excluded from the feature variables for the reason that the male : female ratio is not significantly different between HBV with LC and HBV without LC ( Table 1). After gender was added to the prediction model as one feature, we did not obtain better results. Meanwhile, the gender feature got lower score for feature selection using the SVM strategy.

Classifier performance depends greatly on the characteristics of the data to be classified. There is no single classifier that works best on all given problems. Therefore, in this study, five supervised machine learning methods were employed to construct classifiers for predicting LC. First, we compared their classification accuracy on the training set by 10-fold cross-validation. The ACCs were 93.8%, 97.5%, 90.1%, 86.4%, and 85.2% for LC-NB, LC-MLP, LC-SVM, LC-DT, and LC-CART, respectively. Because a subset of features that have high classification accuracy on the training set may not have good generalization properties [35], we thus applied our trained classifiers to the test set to avoid biases. The fact that the small decrease in accuracy was obtained on the test set demonstrates the generalization performance of the classifiers. Among the five classifiers, the AUCs of LC-NB and LC-MLP were better. Meanwhile, the difference of AUCs between LC-NB and LC-MLP has no statistical significance (*P* = 0.7871). As a result, both of them were selected as our prediction classifiers in this study (Table 4 and Figure 3).

When we compared the results of the aforementioned studies with that of this study, we found that no protein/peptide peak can be identified with same molecular mass. Possible reasons for this contradiction may lie in the following aspects. First, prefractionation strategies and detection technology were used in different studies. Second, different statistical and computational methods may lead to the variability. Third, both the patients infected with HBV and healthy individuals were included in the control group in this study.

As is known to all, analytical reproducibility is a significant challenge in MALDI protein profiling [36]. Differences in reagents and handling and changes in room temperature, pressure, and humidity may influence the (co-)crystallization step and cause day-to-day variation [37, 38]. To improve the analytical performance, we standardized the collection and fractionation protocol and optimized parameters of MALDI-TOF MS. In this study, the reproducibility tests showed that the CVs of Autoflex were similar to those in other reports [26, 39]. 

## 5. Conclusions

In conclusion, we describe two classifiers, LC-NB and LC-MLP, based on the serum peptide pattern for prediction of HBV-infected LC from MALDI-TOF MS in this study. The higher accuracy of the LC-NB and LC-MLP suggests that they have a great potential to emerge as noninvasive approaches in the screening of LC. Consideration has been made for further verification of their accuracy, sensitivity, and specificity in larger population from different regions and different age ranges.

## Figures and Tables

**Table 1:** Participant demographics.

	CHB cirrhosis			Control						Total
	HBV with LC			HBV without LC			Healthy individuals		
	Training	Test	Total	Training	Test	Total	Training	Test	Total
Total number of patients	22	22	44	23	23	46	36	36	72	162
Mean (range) age in years	49.28 (25–68)	51.75 (32–71)	50.16 (25–71)	47.83 (28–59)	46.39 (28–61)	47.11 (28–64)	47.08 (35–55)	47.97 (32–56)	47.55 (32–56)	48.12 (25–71)
Sex (male : female)	16 : 6	17 : 5	33 : 11	15 : 8	17 : 6	32 : 14	19 : 17	18 : 18	37 : 35	102 : 60

**Table 2:** Top twenty peptide patterns selected between each among the three groups.

HBV with LC versus HBV without LC	HBV with LC versus healthy individuals	HBV without LC versus healthy individuals
3880	4165	2670
6945*	6945*	4165
4202*	4133	4061
4267	2670	4298
2929	4465	6451
807*	1928*	1449*
3889	916*	4207*
3027	1536*	3260
8946	4207*	2929
6451	4281*	6636
1017*	1011*	8946
1531	1449*	876
2080	853	2551*
1942	4789	5906
6974	1933	3339
6649	2551*	3951*
1933	2080	2035
4281*	1531	6649
1043	860	4119
1785*	807*	4353

*Represents peptide in the selected peptide pattern.

**Table 3:** Comparison of performance via tenfold cross-validation.

Classifiers	TP	TN	FP	FN	ACC (%)	SE (%)	SP (%)
LC-NB	19	57	2	3	93.8	86.4	96.6
LC-MLP	21	58	1	1	97.5	95.5	98.3
LC-SVM	16	57	2	6	90.1	72.7	96.6
LC-DT	15	55	4	7	86.4	68.2	92.7
LC-CART	13	56	3	9	85.2	59.1	94.9

**Table 4:** Performance of the five classifiers on the test set.

Classifiers	TP	TN	FP	FN	ACC (%)	SE (%)	SP (%)	AUC
LC-NB	19	57	2	3	93.8	86.4	96.6	0.977
LC-MLP	20	56	3	2	93.8	90.9	94.9	0.973
LC-SVM	17	55	4	5	88.9	77.3	92.7	0.853
LC-DT	17	53	6	5	86.4	77.3	89.8	0.825
LC-CART	14	48	11	8	76.5	63.6	81.4	0.733

## Abbreviations

HBV: Hepatitis B virus
CHB: Chronic hepatitis B
SVM: Support vector machine
HCC: Hepatocellular carcinoma
ARFI: Acoustic radiation force impulse
CV: Coefficient of variance
WEKA: Waikato Environment for Knowledge Analysis
TP: True positive
TN: True negative
FP: False positive
FN: False negative
SE: Sensitivity
SP: Specificity
ACC: Accuracy
ROC: Receiver operator curve
ANN: Artificial neural network.
